# Soluble amyloid composition links proximal APP processing, fibrillar burden and the p-tau181 response

**DOI:** 10.21203/rs.3.rs-10955863/v1

**Published:** 2026-10-22

**Authors:** Norberto Rodriguez-Espinosa

## Abstract

Amyloid biomarkers sample distinct levels of an open, kinetically coupled biochemical system, yet scalar summaries may obscure biologically relevant configuration. We asked whether separating shared soluble-peptide variation from Aβ42-selective depletion preserves information and clarifies the amyloid context of the soluble tau response. In 380 Alzheimer’s Disease Neuroimaging Initiative participants, CSF Aβ38, Aβ40 and Aβ42 mass-spectrometry measurements were used to derive two peptide-only analytical constructs: variation shared by Aβ38 and Aβ40 (T), and Aβ42-selective depletion relative to their joint profile (D42). BACE activity and sAPPβ preferentially aligned with T (incremental-R² separation 0.463; 95% bootstrap interval 0.361–0.535). D42 was preferentially associated with later fibrillar burden: among 91 participants with PET a median 4.98 years later, the D42–T contrast was 31.21 Centiloids/SD (95% CI 11.12–51.30). In 375 participants, p-tau181 was independently associated with both constructs (standardized coefficients 0.518 and 0.472), mapping across the T–D42 state space and revealing a supported region of elevated soluble tau. Information-retention analyses showed that tested scalar compressions discarded readout-relevant information. Together, these findings support a two-coordinate representation of soluble amyloid state that preserves biologically relevant information obscured by scalar summaries and locates the soluble tau response within the joint peptide configuration.

